# Binding of MutS protein to oligonucleotides containing a methylated or an ethylated guanine residue, and correlation with mutation frequency

**DOI:** 10.1016/j.mrfmmm.2007.12.009

**Published:** 2008-04-02

**Authors:** Kentaro Taira, Shintaro Nakamura, Khota Nakano, Daisuke Maehara, Keinosuke Okamoto, Sakae Arimoto, David Loakes, Leroy Worth, Roel M. Schaaper, Kohji Seio, Mitsuo Sekine, Kazuo Negishi, Tomoe Negishi

**Affiliations:** a Graduate School of Medicine, Dentistry and Pharmaceutical Sciences, Okayama University, Tsushima, Okayama 700-8530, Japan; b MRC, Laboratory of Molecular Biology, Hills Road, Cambridge CB2 2QH, United Kingdom; c NIEHS, Research Triangle Park, NC 27709, United States; d Department of Life Science, Faulty of Bioscience and Biotechnology, Tokyo Institute of Technology, Nagatsuda, Nidoriku, Yokohama 226-8501, Japan; e Gene Research Center, Okayama University, Tsushima, Okayama 700-8530, Japan

**Keywords:** MutS, Mismatch repair, *O*^6^-methylguanine, *O*^6^-ethylguanine, Mutation

## Abstract

The MutS-based mismatch repair (MMR) system has been conserved from prokaryotes to humans, and plays important roles in maintaining the high fidelity of genomic DNA. MutS protein recognizes several different types of modified base pairs, including methylated guanine-containing base pairs. Here, we looked at the relationship between recognition and the effects of methylating versus ethylating agents on mutagenesis, using a MutS-deficient strain of *E. coli*. We find that while methylating agents induce mutations more effectively in a MutS-deficient strain than in wild-type, this genetic background does not affect mutagenicity by ethylating agents. Thus, the role of *E. coli* MMR with methylation-induced mutagenesis appears to be greater than ethylation-induced mutagenesis. To further understand this difference an early step of repair was examined with these alkylating agents. A comparison of binding affinities of MutS with *O*^6^-alkylated guanine base paired with thymine, which could lead to transition mutations, versus cytosine which could not, was tested. Moreover, we compared binding of MutS to oligoduplexes containing different base pairs; namely, *O*^6^-MeG:T, *O*^6^-MeG:C, *O*^6^-EtG:T, *O*^6^-EtG:C, G:T and G:C. Dissociation constants (*K*_d_), which reflect the strength of binding, followed the order G:T- > *O*^6^-MeG:T- > *O*^6^-EtG:T- = *O*^6^-EtG:C- ≥ *O*^6^-MeG:C- > G:C. These results suggest that a thymine base paired with *O*^6^-methyl guanine is specifically recognized by MutS and therefore should be removed more efficiently than a thymine opposite *O*^6^-ethylated guanine. Taken together, the data suggest that in *E. coli*, the MMR system plays a more significant role in repair of methylation-induced lesions than those caused by ethylation.

## 1 Introduction

The MutS-based mismatch repair (MMR) system is important for recognition and removal of mismatched base pairs and small insertion/deletion loops during DNA replication, and in maintaining the high fidelity of genomic DNA by preventing the accumulation of mutations [reviewed by 1–3]. MMR pathway components such as MutS-related proteins have been widely conserved from prokaryotes to eukaryotes. Additionally, deficiency in MMR pathway components increases both the rate of spontaneous mutation and the rate of mutation induced by UV light [4] or alkylating agents [5–7], and has also been implicated in carcinogenesis, including hereditary nonpolyposis colon cancers [8] and skin cancers [9]. MutS protein initiates MMR by binding to mismatched base pairs in DNA. MutS protein can also bind to sites between normal and damaged bases such as UV photoproducts [10,11], methylated bases [12,13], and base analogues [14,15].

The results of several reports suggest that MutS specifically binds to *O*^6^-methylguanine (*O*^6^-MeG):T base pairs and is responsible for reducing mutations caused by those lesions. Additionally, in eukaryotic cells, binding of MutS to damaged DNA appears to trigger a signaling cascade that leads to DNA strand breaks, cell cycle arrest and apoptosis [16–18]. Alkylating agents produce *O*^6^-alkylguanine and *O*^4^-alkylthymine as major mutagenic DNA lesions. However, it is unclear if *O*^6^-ethylguanine in DNA is a target of MutS and thus, the MMR pathway. In this study, we compared the mutagenic activities of methylating agents to that of ethylating agents in a MutS-negative *E. coli* strain. We also determined if *O*^6^-ethylguanine (*O*^6^-EtG):T and *O*^6^-AlkylG:C base pairs are recognized by *E. coli* MutS protein using a gel shift assay (EMSA). The frequency at which alkylating agent induce mutations was elevated in a MutS-deficient strain, whereas for ethylating agents, we observed no difference in the frequency of mutations induced in MutS-deficient and wild-type strains. Consistent with this, we found that *O*^6^-EtG:T was recognized by MutS less efficiently than *O*^6^-MeG:T.

## 2 Materials and methods

### 2.1 Chemicals

*N*-methyl-*N*-nitrosourea (MNU) (CAS 684–93–5) and *N*-ethyl-*N*′-nitro-*N*-nitroso-guanidine (ENNG) (CAS 63885–23–4) were purchased from Nakarai Tesque (Kyoto, Japan), and ethyl methansulfonate (EMS) (CAS 62–50–0) from Wako Pure Chemicals (Osaka, Japan). *N*-ethyl-*N*-nitrosourea (ENU) (CAS 759–73–9), methyl methansulfonate (MMS) (CAS 66–27–3) and *N*-methyl-*N*′-nitro-*N*-nitrosoguanidine (MNNG) (CAS 70–25–7) were purchased from Sigma–Aldrich Chemicals (St. Louis, MO).

### 2.2 Bacteria strains and mutation assay

The bacterial strains used for detection of mutations were as follows: a wild-type strain, NR10832, containing F′CC102 (F′ *prolac* episome); and a MMR deficient strain, NR12896 (*mutS201*::Tn5, F′CC102). These strains were derived from the parental strain KA796 as described by Negishi et al. [14]. The *lac* allele of F′CC102 reverts only by G·C to A·T transition [19]. An *E. coli lacZ* reversion assay was preformed to detect mutagenicity of alkylating agents in the wild-type or in MMR deficient strain. For mutagenesis, overnight cultures of each strain (0.1 ml) were incubated with the mutagen in 0.1 M sodium phosphate buffer (pH 7.4) for 1 h at 37 °C. Next, 0.1 ml of the treated cultures were spread on lactose agar plates to determine the number of revertants, and an adequately diluted samples were also spread on minimal glucose plates to determine the total variable cell number. Mutational frequencies were calculated by dividing the number of revertant colonies by the number of total colony-forming cells on glucose plates. Statistical analysis was performed using the Student's *t*-test.

### 2.3 Oligonucleotides

The oligonucleotide duplexes used in this study are shown in Table 1. The oligonucleotides KN-1, 2 and 3 (HPLC-purified grade) were purchased from Greiner Bio-One Japan (Tokyo, Japan). In addition, KNG-1and KNG-2were synthesized and purified via SDS-PAGE (KNG-1) or HPLC (KNG-2) [20]. This sequence was derived from the active site of the *E. coli lacZ* gene. To prepare 5′-^32^P-labeled oligonucleotides, KN-2 and KN-3 were labeled with T4 polynucleotide kinase (Takara-bio, Kusatsu, Japan) and [γ-^32^P] ATP, followed by removal of unincorporated [γ-^32^P] ATP using G-25 Sephadex Quick-Spin Column (Boehringer Mannheim, Indianapolis, IN). 0.2 μM Each labeled oligomer (KN-2 and KN-3) was combined with 0.4 μM corresponding unlabeled complementary strand (KN-1, KNG-1 or KNG-2) in annealing buffer (10 mM sodium phosphate buffer (pH 7.4), 50 mM NaCl and 1 mM EDTA), heated to 95 °C for 3 min, incubated at 65 °C for 15 min, and then annealed for 2–3 h. The duplexes were then mixed with NaCl and benzoylated naphthoylated DEAE-cellulose (Sigma Co.), which binds to single strand-oligonucleotides, and the duplexes were eluted from a G-25 Sephadex Quick-Spin Column (single strand oligomers remain in the column).

### 2.4 Purification of MutS protein

**Fig. 1:**
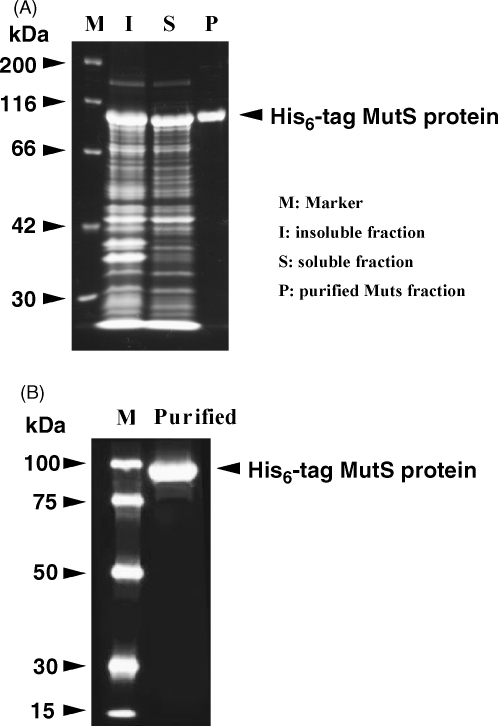
The purity of MutS protein used in this study shown by Coomassie blue staining (A) and Western blotting (B) of SDS-PAGE.

His_6_-tagged MutS protein was purified as described by Worth et al. [21], using the QIAexpressionist kit (Qiagen). Briefly, M15pREP4 cells transformed with pLW10, a MutS expression plasmid, were grown. Next, production of recombinant MutS protein was induced with IPTG. Cells were then sonicated and the soluble and insoluble fractions were separated. MutS protein was prepared from the soluble fraction using the TALON Metal Affinity Resin (Clontech) according to the manufacturer's protocol. Purity of the His_6_-tagged MutS protein was determined by SDS-PAGE with Coomassie blue staining, followed by immuno-blotting analysis and detection via the Anti-His HRP Conjugate Kit (Qiagen) as shown in Fig. 1. Purified MutS protein was stored at −80 °C (50 mM Hepes pH 7.0, 200 mM KCl, 1 mM DTT, 1 mM EDTA and 13.3% glycerol) until its use in the Gel shift assay EMSA.

### 2.5 Gel shift assay

A gel shift assay was used to estimate the affinity of MutS protein to an oligonucleotide containing a modified guanine residue. Each labeled and purified oligonucleotide (2 pmol) was incubated with 0–520 nM MutS protein for 15 min at 37 °C in band shift buffer (20 mM Tris–HCl; pH 7.6; 50 mM KCl; 5 mM MgCl_2_; 1 mM dithiothreitol; 0.05 mg/ml BSA) in 20 μl. Each reaction contained 20 pmol of unlabeled GC duplex. After incubation, MutS-oligonucleotide complexes were separated by electrophoresis on a 5% non-denaturing polyacrylamide gel and analyzed using a Bio-Imaging analyzer (BAS2000, Fuji Photo Film, Tokyo).

## 3 Results

### 3.1 Mutagenic activity of alkylating agents

**Fig. 2:**
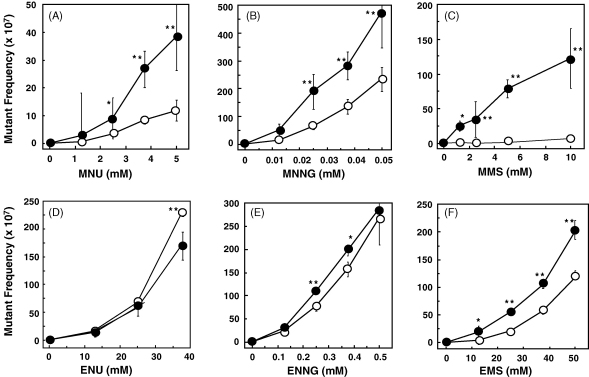
The mutational frequencies of MNU (A), MNNG (B), MMS (C), ENU (D), ENNG (E) and EMS (F) in a MutS-deficient strain (filled circle) or wild-type (open circle). *P < 0.05, **P < 0.01 compared to the mutant frequency for the wild-type. Three to six independent experiments were performed for each. Mutational frequencies were obtained after correcting for the frequency of spontaneous mutations. The frequencies of spontaneous mutations are (34.3 ± 3.3) × 10^−7^ for the MutS (−) strain (NR12986) and (0.3 ± 0.1) × 10^−7^ for the wild-type strain (NR10832).

To evaluate the involvement of MMR in protection against mutagenesis induced by DNA methylation and ethylation, the mutagenicity of MNU, MNNG, MMS, ENU, ENNG or EMS was measured in repair proficient- and MutS-deficient *E. coli* CC102 cells, in which GC to AT reversions can be detected. For both strains, the mutational frequency increased with an increase in the concentration of each alkylating agent applied to the cells. The mutational frequencies for MNU, MNNG and MMS were elevated in the MutS-deficient strain more sharply than in wild-type (Fig. 2A, B and C). In contrast, the mutational frequencies induced by ENU and ENNG were almost the same between the strains (Fig. 2D and E). The frequencies of mutation by EMS were significantly higher in MutS (−) than that in wild-type, but the differences were very small. Treatment of cells with mutagens at these concentrations did not result in significant levels of cell death.

### 3.2 Binding specificity of MutS protein

**Fig. 3:**
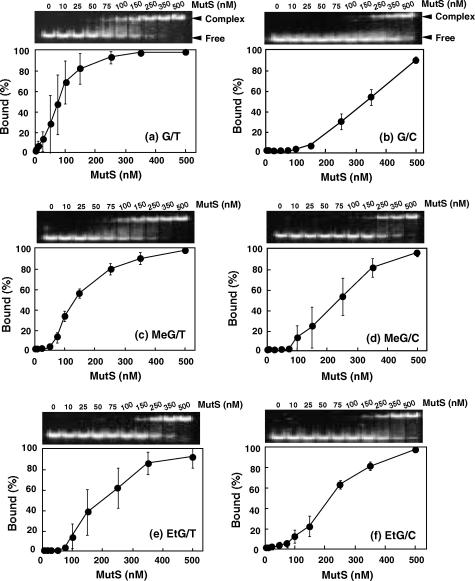
Autoradiograms of gel shift assay and binding affinity of *E. coli* MutS protein bound with each of six different oligoduplexes, which contained either *O*^6^-MeG:T, *O*^6^-MeG:C, *O*^6^-EtG:T, *O*^6^-EtG:C, G:T or G:C base pair. Each 5′-^32^P-labeled duplex was incubated with purified MutS and subjected to native polyacrylamide gel electrophoresis. The percent of duplexes binding to MutS was calculated by dividing the densities of bands showing complexes by total densities of all labeled-bands at each concentration of MutS. The concentrations of MutS used were (left to right) 0, 10, 25, 50, 75 100, 150, 250, 300 and 500 nM.

The difference in the effects of MutS deficiency on the outcomes of mutagenesis induced by methylating or ethylating agents suggests that there may be different affinities of the protein toward base pairs containing methylated versus ethylated DNA. Indeed, we find that MutS protein binds more tightly to *O*^6^-MeG:T than to *O*^6^-EtG:T pairs. Using a gel shift assay, we examined binding of MutS protein to each of six duplexes, which contained *O*^6^-MeG:T, *O*^6^-MeG:C, *O*^6^-MeG:T, *O*^6^-EtG:T, *O*^6^-EtG:C, G:T or G:C pair (Table 1). As shown in Fig. 3, the band corresponding to MutS-bound to the *O*^6^-MeG:T duplex was clearly observable at 75 nM of MutS. However, the oligonucleotide duplex containing *O*^6^-EtG:T has a weaker affinity to MutS than the *O*^6^-MeG:T duplex. The band corresponding to MutS in a complex with the *O*^6^-EtG:T duplex was only seen at MutS concentrations of more than 150 nM. The *O*^6^-MeG:C and *O*^6^-EtG:C duplexes showed similar affinities to MutS. The percentage of MutS-bound duplex was determined after gel separation (Fig. 3). The dissociation constant (*K*_d_) of each complex was obtained by determining the concentration at which half the duplexes were bound to MutS. The affinity of MutS to oligonucleotide followed the order G/T > *O*^6^-MeG:T > *O*^6^-EtG:T ≥ *O*^6^-EtG:C = *O*^6^-MeG:C pairs, and *K*_d_ values were 75, 120, 210, 220, and 220 nM, respectively. This suggests that among base pairs containing alkylated guanines, the *O*^6^-MeG:T base pair in DNA can be recognized and repaired efficiently.

## 4 Discussion

Several lines of evidence suggest that the MMR pathway components are involved in repair of several types of DNA lesions, including those induced by alkylating agents, which result primarily in the mutagenic lesion *O*^6^-alkylguanine. Among modified DNA bases, *O*^6^-methylguanine has been demonstrated to be recognized by MutS [12,13]. In contrast, little is known about whether or not *O*^6^-ethylguanine is also a substrate for MMR pathway components, although there is a report that the mutagenicity of ENU is higher in an Msh2 deficient mouse cell line [7]. In the present study, we compared the effects of different alkylating agents in a genetic background deficient in MMR.

The results suggest that ethylated bases are less readily recognized by the MMR system than methylated bases. The frequency of mutations in a MutS-deficient *E. coli* strain treated with ENU was similar to what was observed for a wild-type strain, whereas MNU-induced mutation was at a higher frequency in MutS-deficient cells than in wild-type. ENNG and MNNG showed similar responses in MutS-deficient and proficient cells to ENU and MNU, respectively. The mutations induced by alkyl-methansufonates, EMS and MMS, increased more efficiently by the MutS deficiency than other alkylating agents. *N*-alkyl-nitrosourea and *N*-alkyl-nitrosoguanidine compounds produce *O*^6^-alkylguanine via the SN1 reaction more efficiently than alkyl-methansufonates through the SN2 reaction [22,23]. The mutation inducibilities of alkyl-methansufonates might be more influenced by MMR due to lower yields of *O*^6^-alkylguanine.

Based upon this differential reactivity and the results presented here, we hypothesize that *E. coli* MutS protein binds more tightly to *O*^6^-methylguanine than to *O*^6^-ethylguanine, and that this affinity results in more efficient repair of *O*^6^-methylguanine than that of *O*^6^-ethylguanine. We demonstrated that this is true when the target sequence is CC102, where the GC-to-AT reversion can be readily detected. The mutagenic process by which a GC pair is converted to an AT pair starts with incorporation of thymine opposite *O*^6^-methylguanine. In MMR proficient cells, recognition by MutS will result in efficient removal of the thymine by the MMR system, effectively suppressing mutagenesis. In contrast, the relatively inefficient recognition of *O*^6^-EtG:T by MutS seems to a the *O*^6^-EtG:T base pair to persist, eventually resulting in mutation even in MutS-proficient or MutS-deficient strains. When the modified guanine residue correctly pairs with cytosine, the resultant base pair is poorly recognized by MutS, and thus, the cell avoids repairing correctly paired bases, even in the presence of a methylated or ethylated guanine.

Recently, Yoshioka et al. reported that MutSα, which is a heterodimer of mammalian MutS homologues, binds to the *O*^6^-MeG:T base pair to the same extent that it binds to G:T but does not bind effectively to *O*^6^-MeG:C or G:C base pairs [16]. Thus, the human MutSα appears to be more selective than its *E. coli* homologue, distinguishing between the mutagenic *O*^6^-MeG:T and the inconsequential *O*^6^-MeG:C. It is thought that accumulation of *O*^6^-MeG:T base pairs can lead to cell death or apoptosis, as *O*^6^-MeG in template DNA induces reiterative repair attempts via the MMR system, a process termed futile cycling, followed by DNA strand breaks [22,24]. Yoshioka et al. have also demonstrated that in HeLa cells, the presence of *O*^6^-MeG-DNA adducts initiates ATR checkpoint signaling through MMR, which leads to cell cycle arrest and apoptosis [16]. Moreover, Armstrong and Galloway have studied the genotoxicity of methylating and ethylating agents in mammalian cells with and without a functional MMR system. Their data indicate that *O*^6^-MeG:T base pairs induce chromosome aberrations at lower doses of methylators in MMR(+) cells than in MMR(−) cells, whereas *O*^6^-EtG:T base pairs do not have a similar effect [25]. Wyatt and Pittman reported that methylated base triggered DNA strand breaks [26]. Previously, we found that in a Drosophila somatic mutation assay, which primarily detects recombination events, the mutagenicity of the methylating agent *N*-nitrosodimethylamine (NDMA) is 30 times higher than that of the ethylating agent *N*-nitrosodiethylamine (NDEA). We also found that the levels of *O*^6^-alkyldeoxyguanosine in DNA were similar after cells were exposed to the same dose of NDMA or NDEA. We concluded that a methylated base could induce recombinational events in the chromosome more often than ethylated bases [27]. This suggests that *O*^6^-MeG residues may be more efficient at inducing futile cycling than *O*^6^-EtG. These results suggest that eukaryotic MutS homologues also bind *O*^6^-MeG:T better than they bind to *O*^6^-EtG:T; however, this remains to be addressed by direct experimentation.

In the present study, we show that *E. coli* MutS protein can recognize *O*^6^-MeG:T more efficiently than *O*^6^-EtG:T base pairs, and that this difference in relative affinities would explain MNU induce mutations versus ENU in MutS-proficient and deficient strains. However, alkylated DNA lesions (sites) are recognized not only by MMR but also via other means, such as the NER and by the alkylguanine-DNA-alkyltransferase (AGT) which could act in concert to rectify incorrect base pairs formed through modified bases [28]. Further investigation will be necessary to help uncover the extent to which MMR is involved in the repair of alkylated DNA lesions. We constructed new strains by introduction of deficiency of AGT or NER into the strain used in this study, and are going to examine the influence to mutagenesis.

## Figures and Tables

**Table 1:**
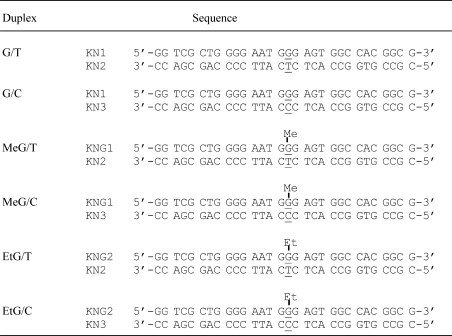
The DNA sequences of oligonucleotide duplexes used in this study
